# Optoacoustic Monitoring of Physiologic Variables

**DOI:** 10.3389/fphys.2017.01030

**Published:** 2017-12-12

**Authors:** Rinat O. Esenaliev

**Affiliations:** Laboratory for Optical Sensing and Monitoring, Department of Neuroscience and Cell Biology, Department of Anesthesiology, Center for Biomedical Engineering, University of Texas Medical Branch, Galveston, TX, United States

**Keywords:** optoacoustic, photoacoustic, monitoring, imaging, sensing, physiologic

## Abstract

Optoacoustic (photoacoustic) technique is a novel diagnostic platform that can be used for noninvasive measurements of physiologic variables, functional imaging, and hemodynamic monitoring. This technique is based on generation and time-resolved detection of optoacoustic (thermoelastic) waves generated in tissue by short optical pulses. This provides probing of tissues and individual blood vessels with high optical contrast and ultrasound spatial resolution. Because the optoacoustic waves carry information on tissue optical and thermophysical properties, detection, and analysis of the optoacoustic waves allow for measurements of physiologic variables with high accuracy and specificity. We proposed to use the optoacoustic technique for monitoring of a number of important physiologic variables including temperature, thermal coagulation, freezing, concentration of molecular dyes, nanoparticles, oxygenation, and hemoglobin concentration. In this review we present origin of contrast and high spatial resolution in these measurements performed with optoacoustic systems developed and built by our group. We summarize data obtained *in vitro*, in experimental animals, and in humans on monitoring of these physiologic variables. Our data indicate that the optoacoustic technology may be used for monitoring of cerebral blood oxygenation in patients with traumatic brain injury and in neonatal patients, central venous oxygenation monitoring, total hemoglobin concentration monitoring, hematoma detection and characterization, monitoring of temperature, and coagulation and freezing boundaries during thermotherapy.

## Introduction

Optical techniques can be used for noninvasive measurements of physiologic variables, functional imaging, and hemodynamics monitoring. These measurements are mostly based on high optical absorption contrast of tissue chromophores (Welch and van Gemert, 2011; Tuchin, 2016). However, optical techniques have drawbacks associated with limited resolution due to strong light scattering in tissues.

Optoacoustic technique utilizes thermoelastic generation of optoacoustic (ultrasound) waves in tissues by short optical pulses. Optoacoustic pressure wave amplitude is linearly dependent on the absorption coefficient. Time-resolved detection of the optoacoustic waves yields high (optical) contrast and high (ultrasound) resolution that can be used for imaging, sensing, and monitoring in tissues.

Since early 1990s we have been working on biomedical optoacoustics and proposed to use it for many diagnostic applications, developed and built optoacoustic systems, and tested them in tissues phantoms, tissues *in vitro* and *in vivo*, animal models, and clinical studies (Esenaliev et al., 1993, 1996, 1997, 1998a, 1999b, 2002a, 2004a,b; Oraevsky et al., 1998; Larin et al., 2001, 2002, 2005; Larina et al., 2005; Petrova et al., 2005, 2009; Petrov et al., 2005, 2006, 2012a,b, 2014, 2016, 2017a,b; Brecht et al., 2007; Patrikeev et al., 2007; Herrmann et al., 2017). In this review we present origin of contrast and high spatial resolution in physiologic measurements and discuss results obtained with the optoacoustic systems *in vitro*, in animals, and in humans on monitoring of physiologic variables.

## Origin of high contrast and resolution in optoacoustic measurements

### Theoretical background

The thermoelastic mechanism of optoacoustic wave generation is based on absorption of light energy in a medium followed by temperature rise and thermal expansion in the medium. The thermal expansion of the irradiated medium induces mechanical stress (pressure rise). A short optical pulse with the incident fluence, F_o_, induces a pressure rise, P(z), in the medium upon condition of stress confinement:

(1) $$\begin{array}{r} P\left(z\right)=\left(\beta {c_{s}}^{2}/C_{p}\right)\mu_{a}F=\Gamma \mu_{a}F\left(z\right)=\Gamma \mu_{a}F_{o}\exp\left(-\mu_{a}z\right) \end{array}$$

where β [1/°C] is the thermal expansion coefficient; c_s_ [cm/s] is the speed of sound; C_p_ [J/g°C] is the heat capacity at constant pressure; F(z) [J/cm^2^] is the fluence of the optical pulse; and μ_a_ [cm^−1^] is the absorption coefficient of the medium. The generated optoacoustic pressure can be expressed in J/cm^3^ or in bar (1 J/cm^3^ = 10 bar). The combination of the thermophysical parameters, β${\text{c}}_{\text{s}}^{2}$/C_p_ in Equation (1) represents the Grüneisen parameter, Γ (dimensionless). The exponential light attenuation in the medium is represented by exp(−μ_a_z).

The Equation (1) is valid upon the stress-confinement condition when pressure relaxation is negligible during the heat deposition. The stress-confined condition is satisfied when light pulse duration, τ_*p*_, is shorter than the stress relaxation time in the irradiated volume,τ_*str*_:

(2) $$\begin{array}{r} \tau_{p}<\tau_{str}=\frac{1}{\mu_{a}c_{s}} \end{array}$$

Nanosecond pulses can be used to generate conditions of stress confinement for many biomedical optoacoustic applications including monitoring of physiologic variables (Esenaliev et al., 1993, 1996, 1998a; Larin et al., 2001, 2002, 2005; Larina et al., 2005; Petrova et al., 2005, 2009; Petrov et al., 2005, 2006; Brecht et al., 2007).

According to Equation (1), optoacoustic pressure amplitude is proportional to the Grüneisen parameter, fluence, and absorption coefficient of the medium, while the pressure spatial profile is dependent on the absorption coefficient. Since z and t are related by the simple equation:

(3) $$\begin{array}{r} z=c_{s}t \end{array}$$

the spatial distribution of optoacoustic pressure P(z) is detected by a wide-band acoustic transducer as a temporal profile P(t):

(4) $$\begin{array}{r} P\left(t\right)=\Gamma \mu_{a}F_{o}\exp\left(-\mu_{a}c_{s}t\right) \end{array}$$

Therefore, by analyzing the amplitude and/or temporal profile of optoacoustic waves, one can measure the absolute value of the absorption coefficient of the irradiated medium.

Most tissues are strongly scattering media in the optical spectral range. In addition to the absorption coefficient, two other major optical parameters are responsible for distribution of light in tissues: scattering (μ_s_) and effective attenuation (μ_eff_) coefficients. Attenuation of diffusively scattered light depends on the effective attenuation coefficient which is related to μ_a_, μ_s_, and the anisotropy factor (g) as:

(5) $$\begin{array}{r} \mu_{eff}={\left\{3\mu_{a}\left[\mu_{a}+\mu_{s}\left(1-g\right)\right]\right\}}^{1/2} \end{array}$$

where μ_s_(1–g) is the reduced scattering coefficient, μ^s^′ (Welch and van Gemert, 2011). Light penetration depth in tissues is defined as 1/μ_eff_. Distribution of laser fluence and, therefore, pressure in tissue (not very close to the surface) is dependent on optical absorption and effective attenuation coefficients:

(6) $$\begin{array}{r} P\left(z\right)=\Gamma \mu_{a}kF_{o}e^{\left(-\mu_{eff}z\right)} \end{array}$$

where *k* is the parameter resulted from multiple scattering in tissue and depends on absorption and scattering coefficients (Welch and van Gemert, 2011).

Absorption and reduced scattering coefficients of tissues are low in the near-IR spectral range (from 700 to 1,300 nm), that results in deeper penetration of near-IR radiation compared with that of other parts of the spectrum. Application of near-IR radiation allows for sufficient penetration of light in tissues for optoacoustic measurements of physiologic variables.

### Monitoring of temperature

Accurate temperature mapping with sub-mm spatial resolution may provide precise thermotherapy of abnormal tissues with minimal damage to surrounding normal tissues. Amplitude of optoacoustic pressure waves induced in many tissues increases with temperature, mostly due to temperature dependence of the thermal expansion coefficient (Esenaliev et al., 1998b; Larin et al., 2002, 2005; Larina et al., 2005). The temperature-dependent Grüneisen parameter can be expressed with an equation:

(7) $$\begin{array}{r} \Gamma =A+BT \end{array}$$

where A and B are constants and T is temperature. One can modify (Equation 1) for the case of absorbing media without scattering as:

(8) $$\begin{array}{r} P\left(z\right)=\left(A+BT\left(z\right)\right)\mu_{a}F_{o}e^{\left(-\mu_{a}z\right)} \end{array}$$

and for the case of strongly scattering media in deeper (not in the subsurface) areas as:

(9) $$\begin{array}{r} P\left(z\right)=\left(A+BT\left(z\right)\right)k\mu_{a}F_{o}e^{\left(-\mu_{eff}z\right)} \end{array}$$

where T(z) is the temperature distribution in tissue. One can rearrange (Equation 9) to obtain temperature distribution in tissue:

(10) $$\begin{array}{r} T\left(z\right)=C+D\cdot P\left(z\right)/P{\left(z\right)}_{\text{T}=\text{To}} \end{array}$$

where P(z)_T = To_ is the optoacoustic pressure profile recorded at the initial temperature T_o_ and C and D are parameters that are dependent on tissue properties. Therefore, by recording and analyzing the temporal optoacoustic pressure profile, one can reconstruct distribution of temperature during hyperthermia.

We experimentally demonstrated linear increase with temperature of optoacoustic pressure amplitude in tissue phantoms and tissues such as liver and myocardium (Esenaliev et al., 1998b). In another set of experiments, using rapid heating, we produced temperature gradients in tissue and tissue-like sample (Larina et al., 2005). During the heating we monitored the temperature distribution with optoacoustic system, while a multi-sensor temperature probe inserted in the samples measured actual temperature distribution. These studies demonstrated that the accuracy of optoacoustic temperature was better than 1°C at the spatial resolution less than 1 mm.

### Monitoring of coagulation and freezing front

Tissue optical and thermophysical properties change due to coagulation or freezing. This may provide fast and accurate optoacoustic feedback during thermotherapy with heating or cooling agents. Because the optoacoustic wave amplitude and temporal parameters are dependent on tissue properties (Equation 6), detection and analysis of the optoacoustic waves during thermotherapy may be used for real-time monitoring of the extent of tissue coagulation or freezing with high resolution and contrast (Esenaliev et al., 1998a; Larin et al., 2002, 2005; Larina et al., 2005).

We performed high-resolution, real-time optoacoustic monitoring of tissue coagulation during conductive heating (Larina et al., 2005) or interstitial heating by CW laser light (Larin et al., 2005). Analysis of optoacoustic signal slopes was used for monitoring tissue heating and dimensions of coagulation lesions. Coagulation was induced in liver, myocardium, and prostate by interstitial CW Nd:YAG laser irradiation of the samples or by conductive heating. The optical properties did not change up to the coagulation temperature (about 53°C), but sharply increased during heating up to 70°C. The interstitial coagulation front was monitored in freshly excised canine tissues in real time with spatial resolution of about 0.6 mm. These results suggested that this technique may be used for real-time precise thermotherapy of malignant and benign lesions at depths of the order of centimeter.

Real-time monitoring of cooling and freezing of tissues, cells, and other biological objects with a high spatial and temporal resolution is necessary for selective cryoablation of cancer and benign tumors and for organs, tissues, and other biological objects in medicine and cryobiology. Using liquid nitrogen, we demonstrated that tissue hypothermia and freezing can be monitored with the optoacoustic technique because both amplitude and profile of the optoacoustic waves change with temperature during cooling and freezing (Larin et al., 2002). Sharp increase of the optoacoustic signal slope was detected between −2 and −4°C resulted from the formation of the frozen zone in liver tissue. High spatial resolution (better than 0.5 mm) was obtained in these studies. Such resolution is sufficient for cryoablation monitoring with high precision.

### Monitoring of exogenous dyes and nanoparticles

Optical absorption contrast can be used for monitoring in tissues of exogenous dyes and nanoparticles with high temporal and spatial resolution. Indocyanine green (ICG), an FDA-approved dye for intravenous injections has a high absorption in the near IR spectral range with a maximum at 800–805 nm. Optoacoustic measurements of ICG-produced signal can be useful for monitoring of cardiac output (CO), cardiac index (CI), blood volume (BV), and hepatic function. We measured amplitude and peak-to-peak amplitude of optoacoustic signals induced in whole arterial blood *in vitro* at clinically relevant ICG concentrations. Both amplitude and peak-to-peak amplitude of the signals linearly increased with ICG concentration with high correlation coefficients (*R*^2^ = 0.990 and 0.991, respectively) (Prough et al., 2008). The blood effective attenuation coefficient derived from the optoacoustic signal slopes also increased linearly with ICG concentration with high correlation coefficient of *R*^2^ = 0.986.

Nano- and microparticles that strongly absorb light can be used for photothermal therapy of abnormal tissues including tumors (Esenaliev, 1999a, 2000, 2016a). High sensitivity of the optoacoustic technique to absorption changes provides basis for monitoring of nanoparticle delivery into tumors and for tumor coagulation. We used the optoacoustic technique for real-time monitoring of nanoparticle accumulation in human tumors of nude mice and the nanoparticle-induced laser thermotherapy of these tumors (Esenaliev et al., 2007).

### Monitoring of hemoglobin concentration

Hemoglobin has a high absorption coefficient in the visible and near-IR spectral range (Welch and van Gemert, 2011). We experimentally demonstrated that both the amplitude and spatial distribution of the generated optoacoustic pressure induced in blood are dependent on total hemoglobin concentration [THb] (Esenaliev et al., 2004a,b; Petrova et al., 2005).

High z-axial resolution of the optoacoustic technique permits direct [THb] measurements in blood vessels because the optoacoustic waves induced in blood arrive at the acoustic transducer at the time defined by Equation (3). Since the hemoglobin absorption coefficient is dependent on hemoglobin saturation (i.e., oxygenation), we used the wavelength of 805 nm (isobestic point) where oxy- and deoxygenated hemoglobin have same absorption (Esenaliev et al., 2004a,b; Petrova et al., 2005). This allows for [THb] measurement at any oxygenation.

### Monitoring of oxygenation

One of the most important optoacoustic applications is oxygenation imaging, monitoring, and sensing (Esenaliev et al., 2002a,b). They can be used for diagnostics and management of large populations of patients including those with traumatic brain injury (TBI), circulatory shock, stroke, patients undergoing surgery, anemic patients, neonatal patients, and fetuses during late-stage labor (Petrova et al., 2005, 2009; Petrov et al., 2005, 2006, 2012a,b, 2014, 2016, 2017a,b; Brecht et al., 2007; Esenaliev et al., 2016b; Esenaliev, 2017; Herrmann et al., 2017).

We proposed and developed a noninvasive, optoacoustic diagnostic platform for measurements of oxygenation, hemoglobin concentration, and other important physiological parameters in tissues and specific blood vessels (Petrova et al., 2005, 2009; Petrov et al., 2005, 2006, 2012a,b, 2014, 2016, 2017a,b; Brecht et al., 2007; Prough et al., 2008; Herrmann et al., 2017). Because hemoglobin is a major chromophore in the near IR spectral range and its absorption depends on oxygenation, optoacoustics is suitable for monitoring of these physiologic variables.

## Discussion

In early 1990s we performed first experimental studies on biomedical optoacoustics in tissues and mid 1990s obtained first results on optoacoustic microscopy and demonstrated optoacoustic signal detection in tissues at several centimeters depths that is well beyond the optical diffusion limit (five times greater than the light penetration depth defined as 1/μ_eff_). High-resolution optoacoustic images of tissue phantoms were reconstructed by our group in early 2000s; since then biomedical optoacoustics/photoacoustics has grown tremendously. Recent original papers and reviews by other groups (and references therein) demonstrate remarkable progress in optoacoustic imaging, cancer detection, microscopy, functional imaging in small animals, optoacoustic instrumentation, dual modality optoacoustic/ultrasound imaging, contrast agents development, and other applications (Jaeger et al., 2013; O'Donnell et al., 2013; Su et al., 2013; Daoudi et al., 2014; Ellwood et al., 2014; Yao and Wang, 2014; Taruttis et al., 2015; Bourantas et al., 2016; Cai et al., 2016; Choi et al., 2016).

The results of our studies demonstrated high contrast and high resolution in optoacoustic monitoring, imaging, and sensing of the physiologic variables. The high contrast originates from thermophysical properties (Grüneisen parameter) and/or from optical properties (absorption and effective attenuation coefficient) of tissue. Table 1 shows the physiologic variables that were monitored in our optoacoustic works, origin of contrast, range, accuracy, and precision of monitoring. Combination of the high contrast with high resolution (from microns to sub-mm) yields a promising diagnostic modality that can be used in clinics and biomedical research. Although light attenuation decreases the monitoring accuracy in thick tissues, high signal-to-noise ratio of optoacoustic signals allowed for accurate measurements of these physiologic variables at depth greater than the optical diffusion limit (Table 1 and references therein).

**Table 1:** Physiologic variables that were monitored in our optoacoustics works, origin of contrast, range, accuracy, precision of monitoring, and corresponding references.

**Variable**	**Origin of contrast**	**Range**	**Accuracy**	**References**
Temperature	Γ	−20° to+70°C	< 1°C (<1 mm resolution)	Esenaliev et al., 1998b; Larin et al., 2002, 2005; Larina et al., 2005
Coagulation	μ_a_, μ_eff_, Γ	0–14 mm (extent of coagulation) 52°-70°C	<0.6 mm axial (coagulation front measurement)	Esenaliev et al., 1998a; Larina et al., 2005; Larin et al., 2005
Freezing	μ_a_, μ_eff_, Γ	0–10 mm (extent of freezing) −20° to 0°C	<0.5 mm axial (freezing front measurement)	Larin et al., 2002
Exogenous dyes and nanoparticles	μ_a_, μ_eff_	0–8 mg/dL	<0.5 mg/dL	Esenaliev et al., 2007; Prough et al., 2008
Total hemoglobin concentration	μ_a_, μ_eff_	5–20 g/dL	1 g/dL (0.2 g/dL precision)	Esenaliev et al., 2004a,b; Petrova et al., 2005; Petrov et al., 2017a
Oxygenation	μ_a_, μ_eff_	10–100%	2.8% (1% precision in veins and arteries)	Petrova et al., 2009; Petrov et al., 2014, 2016, 2017a,b

## Conclusion

We proposed to use optoacoustics for imaging, monitoring, and measurements of a number of important physiologic variables and developed optoacoustic systems for these applications. The systems were successfully tested in tissue phantoms, tissues, animals, and human subjects. The obtained data suggest that this technology may be applicable to large populations of patients. We plan to further develop these systems for single measurement, continuous measurement, and monitoring, as well as for 2D, 3D, and 4D imaging of the physiologic variables.

## Author contributions

The author confirms being the sole contributor of this work and approved it for publication.

### Conflict of interest statement

RE is a co-owner of Noninvasix, Inc., a UTMB-based startup that has licensed the rights to optoacoustic technology.
